# Oral manifestation and salivary changes in renal patients undergoing hemodialysis

**DOI:** 10.4317/jced.53215

**Published:** 2017-02-01

**Authors:** Marieh Honarmand, Leila Farhad-Mollashahi, Alireza Nakhaee, Fahimeh Sargolzaie

**Affiliations:** 1 Oral and Dental Disease Research Center, Department of Oral Medicine, School of Dentistry, Zahedan University of Medical Sciences, Zahedan, Iran; 2 Dept. of Biochemistry, School of Medicine, Zahedan University of Medical Sciences, Zahedan, Iran; 3 Dentist, Dental School, Zahedan University of Medical Sciences, Zahedan, Iran

## Abstract

**Background:**

Salivary changes in hemodialysis patients may result in various oral manifestations. This research intended to determine oral manifestations and some salivary markers in hemodialysis patients.

**Material and Methods:**

This cross-sectional study was conducted on 30 hemodialysis patients (the patient group) and 30 healthy individuals (the control group). Saliva urea and calcium levels and pH values of the participants were measured, and oral manifestations such as pale mucosa, xerostomia, halitosis, changes in the sense of taste, increased calculus formation, gingival bleeding, etc. were recorded in the information collection form. The data was analyzed using T-test and chi-square, and *p*<0.05 was considered to be significant.

**Results:**

The mean salivary urea level and pH value in the patient group were significantly higher compared to those of the control group (*P*<0.05), but there were no significant differences between the two groups with respect to salivary calcium. Halitosis, xerostomia, and increased calculus were the most prevalent manifestations, and gum bleeding was the least prevalent among the patients.

**Conclusions:**

Advanced chronic renal insufficiency can increase salivary urea level, pH value, halitosis, xerostomia, and calculus formation, and may cause pale mucosa.

** Key words:**Renal dialysis, biomarkers, oral manifestation, saliva.

## Introduction

There are about 1.8 million patients with end stage renal disease (ESRD) in the world that need to treatment, including hemodialysis, peritoneal dialysis, or transplantation (1). According to study in 2006, about 12 500 Iranian patients with ESRD (48.5%) received hemodialysis (2).

Dialysis treatment leads to systemic changes, oral complications, and changes in salivary flow rate and saliva composition (3-5).

The importance of saliva as a diagnostic fluid has attracted interest in recent years. The advantages of using saliva, which include its easy availability, non-invasiveness, and the close relationship between saliva and serum parameters, have attracted the interest of researchers in saliva as a unique fluid for diagnosing various diseases (6,7).

In research carried out by Kaushik *et al.* (8) to study oral and salivary changes among hemodialysis patients, it was found that 65% of the patients exhibited at least one of the oral manifestations. The mean stimulated and non-stimulated salivary flow rates in these patients were significantly lower than those of the control group. Mansourian *et al.* (9) conducted a study to compare prevalence of oral lesions in kidney transplant and hemodialysis patients and noticed there was at least one intraoral lesion (including xerostomia, aphthous ulcers, squamous papilloma, gingival inflammation, and candidiasis) in 32.2% of kidney transplant and in 8.6% of dialysis patients. The most prevalent manifestation was xerostomia (4.3%) in dialysis patients, while gingival inflammation (1.1%), and candidiasis (2.2%) were of lower prevalence.

It is necessary to have a thorough knowledge of oral manifestations in hemodialysis patients to take necessary precautions for preventing bacteremia and the consequent complications. Considering the increase in the number of dialysis patients in Iran (10), and since no research had been conducted on hemodialysis patients in Zahedan to simultaneously study salivary markers and oral manifestations, it was decided to investigate oral manifestations and some salivary markers (urea, calcium, and pH) in hemodialysis patients.

## Material and Methods

This cross-sectional study was conducted on 30 hemodialysis patients (at the Hemodialysis Ward of Imam Ali Hospital in Zahedan) and 30 healthy individuals (the control group). The inclusion criteria were for the patients to be older than 18 years old, had been under hemodialysis for more than 1 year, and undergoing hemodialysis in three times a week for the period of 2-4 hours.

Exclusion criteria included history of alcohol use, smoking, caffeine consumption during the past 24 hours, and medical problems except kidney disease (such as history of radiotherapy, Sjogren’s syndrome, etc.) and medications use that influenced salivary glands and their secretion.

The control group had no history of drug therapy or systemic diseases. Moreover, they were matched to the patients group with respect to age, gender, and weight. The purpose of this study was explained along with informed consent taken from all the subjects and Ethics Committee of Zahedan University of Medical Sciences approval taken for this study (No.1051).

Patients received saliva collection tubes before being connected to dialysis machines between 9 and 11 in the morning (the best time for saliva collection). They were asked not to eat, drink, or brush their teeth 90 minutes before the saliva samples were taken to prevent any stimulation. The spitting method was employed to collect non-stimulated saliva: the participant poured saliva once every 60 seconds for 5 minutes into the saliva collection tube. The collected saliva samples were kept in sterilized Falcon tubes with their caps in the closed position and were then coded and sent to the Biochemistry Laboratory of Zahedan University of Medical Sciences where they were centrifuged at 3500 rpm for 20 minutes to separate the debris. The transparent supernatant was kept at -20˚C until tests were carried out to measure the markers.

After saliva collection, intraoral examinations were carried out and the information forms were completed. A oral medicine specialist performed the examinations using disposable mirrors under the light of a strong flashlight, while the patients lay on their beds with their heads at a suitable height.

Oral manifestations included uremic breath that could be smelled at a distance of 10 cm from the patients’ mouths (11), pale mucosa, increased calculus formation, petechial and purpura, candidiasis, changes in the sense of taste, and gingival bleeding. On thorough examination of the oral cavity, these manifestations were recorded in the questionnaires.

Xerostomia was investigated objectively using the tongue blade test: adhesion of a standard wooden tongue blade to the buccal mucosa indicates the mucosa is not sufficiently moistened. Xerostomia was also subjectively determined when patients complained of difficulty in chewing, swallowing, speaking, and overdenture attachment (9).

Saliva was thawed to measure the biochemical parameters in the study. Calcium was measured by using the Ocresolphthalein method and urea by using a urease method based on the protocols of Pars Azmoon Co (Iran), and pH by employing an electrical pH meter. The results of these experiments were recorded in the information forms.

The data was analyzed using SPSS 16 and frequency tables and descriptive statistics, including mean and dispersion indices, were employed to describe the data and, finally, in depended sample t-test and chi-square were used for expressing statistical relationships.

## Results

This research intended to determine oral manifestations and some salivary markers in hemodialysis patients. For this purpose, 30 dialysis patients were selected as the patient group and 30 of healthy individuals as the control group. Seventy percent of the participants were male. The average age of the participants was 38.17±16.88 in the patient group and 40.30±18.34 in the control group, and the mean weight was 70.63±15.31 in the patient group and 73.85±18.45 in the control group.

As shown in  Table 1, the mean levels of saliva urea were different in the two groups: 125.8±68.33 in the patient group and 41.22±16.35 mg/dl in the control group, which indicated a significant difference (*p*=0.0001). Furthermore, the mean pH was 8.41±0.76 in the patient group and 7.01±0.31 in the control group, which showed a significant difference between the two groups (*p*=0.042). Moreover, the measured calcium levels were 2.32±1.15 and 2.47±1.33 mg/dl in the patient and control groups, respectively, which showed there were no significant differences between the two groups in this respect (*p*=0.206).

**Table 1:**
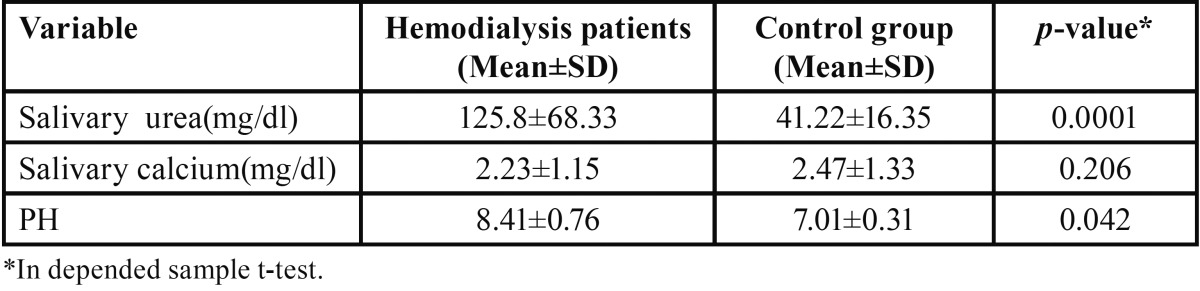
Statistical comparison of mean values between hemodialysis patients and control groups.

 Table 2 indicates halitosis, increased calculus formation, and xerostomia were the most prevalent, and gingival bleeding the least frequent oral manifestations in hemodialysis patients. No cases of pale mucosa or gingival bleeding were observed in the control group.

**Table 2:**
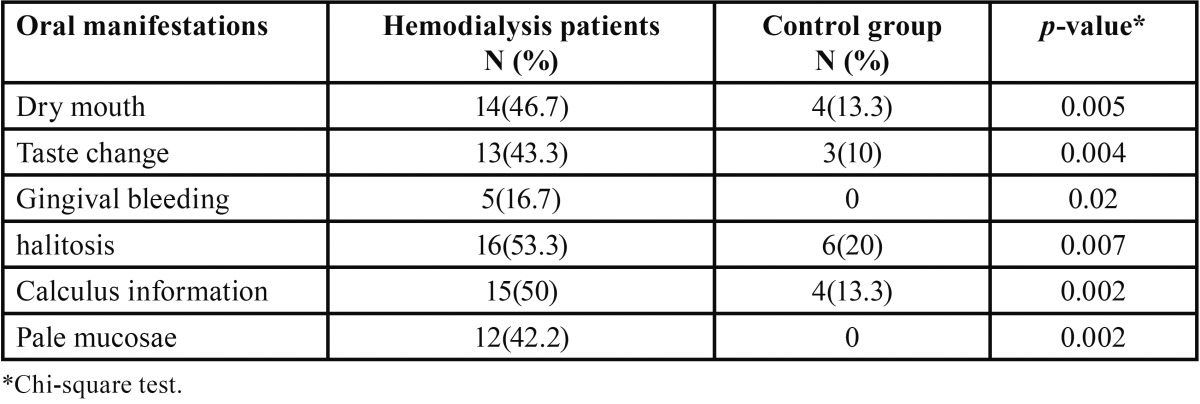
Comparison of oral manifestations between hemodialysis patients and control groups.

## Discussion

This research showed that salivary pH values and urea levels (8.41±0.76 and 125.8±68.33, respectively) in patients with chronic renal insufficiency were higher compared to healthy people, but that there were no significant differences between the two groups in mean salivary calcium levels. As for clinical oral manifestations, halitosis, xerostomia, and increased calculus formation were most prevalent, and gingival bleeding was the least prevalent, among hemodialysis patients.

Increases in urea compounds have been mentioned as one of the findings in most studies carried out on renal patients (12,13). Based on this research, salivary pH values in hemodialysis patients were significantly higher compared to the healthy group (8.41±0.76 versus 7.01±0.31), and these results are in agreement with that found in research conducted by Al Nowaiser *et al.* (14). Salivary urea is decomposed into ammonium ions and carbon dioxide by urease and, hence, may raise salivary pH to critical values (14).

Moreover, high salivary urea levels and decomposition of urea into ammonia increase halitosis in people with kidney diseases. In this research, 53.3% of the patient group and 20% of the control group also suffered from halitosis. Patil *et al.* (15) reported 34% of the patients in their study were afflicted with halitosis. Another reason for increased rates of halitosis could be negligence in oral hygiene because of the chronic nature of the disease in these people (8).

Calcium levels in the patient group were lower than those of the control group, but these differences were not statistically significant. Some studies have referred to reduced calcium levels in hemodialysis patients (12). Chronic uremia is characterized by decreased levels of active metabolites of vitamin D synthesized in the kidneys. The consequence is an increased synthesis and secretion of parathyroid hormone (secondary hyperparathyroidism) causing to the low levels of calcium (16).

The prevalence of xerostomia in the patient group (46.7%) was significantly higher than that of the control group (13.3%). This figure is lower than those reported in study conducted by Patil *et al.* (15) (91%) but almost similar to the 43% reported by Kaushik *et al.* (8). The differences between our results and this reported by Patil *et al.* (15) could probably be due to differences between the studied populations (inclusion and exclusion criteria) and the different methods used in evaluating xerostomia. Xerostomia may be caused by reduced salivary flow rate secondary to atrophy and fibrosis of the salivary glands, taking special drugs, and limited intake of liquids, increasing age, and oral breathing secondary to Pulmonary conditions (17).

Some of the hemodialysis patients experienced changes in the sense of taste (43.3% in the patient group compared to 10% in the control group), and this figure is similar to those reported by Patil *et al.* (15) and Bots *et al.* (18) in their studies. Changes in the sense of taste may have various reasons such as increased levels of salivary urea and dimethyl and trimethylamine levels, metabolic disorders, taking medications, reduced number of taste buds, changes in salivary flow rate and saliva composition in uremic patients (8).

Pale mucosa was another manifestation observed in the patient group (42.2%), while no cases of it were seen in the control group. In study carried out by Patil *et al.* (15) references were made to pale mucosa. Pale mucosa results from anemia mainly developed following the inability of the failing kidneys to secrete erythropoietin, loss of red blood cells through dialysis, increased brittleness of red blood cells and their early destruction and, in some cases, from malnutrition (13).

Increased calculus formation was observed in 50% and 13.3% of the patient and control groups, respectively. In the study conducted by Martins *et al.* (7) calculus was formed in 75% and 36% of the patient and control groups, respectively. Increased salivary urea levels and reduced saliva production can facilitate plaque formation (19).

Gingival bleeding in 16.7% of the members in the patient group was another finding of this study, but no cases of it in the control group. Unnatural bleeding is one of the problems associated with dialysis. Intrinsic platelet abnormalities and impaired plate-let-vessel wall interaction are factor responsible for bleeding tendencies in ESRD. Anemia, dialysis, the accumulation of medications due to poor clearance, and anticoagulation used during dialysis have some role in causing bleeding in ESRD patients (20).

Findings of this research indicate that hemodialysis patients are at greater risk of developing oral manifestations, and that it is necessary that these patients be under careful supervision with respect to oral and dental hygiene and mucosal manifestations. Moreover, timely diagnosis and treatment of oral manifestations will substantially help improve their life satisfaction.
